# Perceptions and utilization of the anti-malarials artemether-lumefantrine and dihydroartemisinin-piperaquine in young children in the Chikhwawa District of Malawi: a mixed methods study

**DOI:** 10.1186/s12936-014-0528-8

**Published:** 2015-01-21

**Authors:** Victoria L Ewing, Dianne J Terlouw, Andrew Kapinda, Cheryl Pace, Esther Richards, Rachel Tolhurst, David G Lalloo

**Affiliations:** Malawi-Liverpool-Wellcome Trust Clinical Research Programme, Queen Elizabeth Central Hospital, Blantyre, Malawi; Liverpool School of Tropical Medicine, Pembroke Place, L3 5QA Liverpool, UK

**Keywords:** Malaria, Antimalarials, ACT, Adherence, Utilization, Perceptions, Malawi, Sub-Saharan Africa, Paediatric

## Abstract

**Background:**

Adherence to anti-malarial dosing schedules is essential to ensure effective treatment. Measuring adherence is challenging due to recall issues and the participants’ awareness of the desired behaviour influencing their actions or responses. This study used qualitative methods, which allow for rapport building, to explore issues around anti-malarial utilization in young children, and used the results to guide the development of a context specific questionnaire on perceptions and adherence to artemether-lumefantrine (AL) and dihydroartemisinin-piperaquine (DHA-PPQ).

**Methods:**

Qualitative data collection included 12 focus group discussions which explored community perceptions of anti-malarials and experiences of administering medications to children. Critical incidence interviews were conducted with 22 caregivers to explore experiences of administering the dispersible or original formulation of AL to young children during recent febrile episodes. A structured questionnaire was used to gather data on experience of recent treatment and adherence to anti-malarials during follow-up visits with 218 caregivers whose child was recently treated with either dispersible AL or DHA-PPQ.

**Discussion/Conclusion:**

Caregivers experience great difficulty in administering medication to children. While the sweet taste of dispersible AL may have reduced conflict between the child and caregiver, sub-optimal dosing due to medication loss remained a problem and overall adherence was greater among those receiving DHA-PPQ, which requires fewer doses. Some caregivers were found to deliberately alter the dosing schedule according to whether they perceived the medication to be too weak or strong. They also developed theories for poor treatment outcomes, such as attributing this to lack of compatibility between the medication and the child. Health education messages should be strengthened to ensure a combination of clear pictorial and verbal instructions are used during dispensing, and consequences of under and over-dosing are explained alongside appropriate responses to possible adverse events. Further optimizing of anti-malarial adherence among children requires the development of anti-malarials with pharmacological properties that allow user-friendly administration and simplified dosing schedules.

## Background

Artemisinin-based combination therapy (ACT) is highly efficacious [1-3], however there are many barriers to ensuring that these drug combinations reach those that need them and that they are used appropriately. Poor adherence to anti-malarial treatment is associated with worsening of symptoms and increased risk of treatment failure and re-infection rates [4-9]. Widespread under-dosing fuels the spread of low-grade drug resistance in a population. Since the introduction of ACT, there has been increasing concern about adherence to anti-malarials. The dosing schedules of ACT is more complex than the previous first-line therapy, sulphadoxine-pyrimethamine (SP) and should resistance to artemisinins become widespread, there are few alternatives.

Adverse events are known to impact on adherence [4,10-13], although ACT has been found to be well-tolerated [1,3,14-16]. Correct understanding of the dosing schedule is essential for adherence [4,10,12,15,17-20]. Difficulty in administering medication to children has been reported by caregivers and shown to impact on adherence [4,18] and caregivers have been found to dislike the required crushing of tablets for young children [21]. The bitter taste of anti-malarials for children is a frequent complaint [18,21] and the full course of medication may not be administered if the child dislikes it [13,22]. Adherence has been found to drop-off towards the end of the treatment regime, even for short-term courses [4,23].

Previous experience in Malawi during the change from chloroquine to SP highlighted the importance of assessing the acceptability of any newly introduced drug [24]. Data on reported adherence must be supplemented with an understanding of why individuals use medications the way they do, in order to appropriately develop interventions to target such issues [25,26]. The Ministry of Health in Malawi is starting to deploy ACT through community health workers operating from rural health posts, starting from so-called hard-to-reach-areas: a good understanding of factors influencing utilization in this context will assist the effective development of such an intervention.

A study was conducted with the aim of exploring, caregiver’s perceptions and experiences of ACT in-depth and understanding how these influenced adherence. Adherence to and perceptions of two different forms of ACT were explored in young children. Two formulations of artemether-lumefantrine (AL) were investigated: the original formulation, which must be crushed for young children, and the newer dispersible formulation which has a sweet cherry flavour. Dihydroartemisinin-piperaquine (DHA-PPQ) is non-dispersible and non-flavoured. In-depth qualitative research was conducted to identify determinants of adherence and inform the development of a structured questionnaire which was then used to gather adherence data in a larger sample.

## Methods

### Study setting

This study was situated within Chikhwawa, a district with high levels of poverty (82% of the population live in poverty and 59% are ultra-poor) [27], situated in the southern region. The public health system in Chikhwawa comprises the district referral hospital, one rural hospital, one mission hospital, 14 Health Centres and 26 Community Health Worker (CHW) operated health posts, which cater for the district’s population of approximately 435,000 [28]. This study was nested within a community-based safety and effectiveness trial investigating the programmatic implementation of AL and DHA-PPQ (Clinical Trial ID No: NCT010380632, http://www.actconsortium.org/projects/18/the-actia-trial-safety-of-repeated-drug-use-in-children). The trial enrolled children between the ages of 4 to11 months, who were randomized at village level to receive either AL or DHA-PPQ from the health facility when malaria was diagnosed, and followed up for up to three years to assess the long term safety of ACT. Malaria in the area of the parent trial shows high heterogeneity seasonally and spatially with village level parasite prevalences ranging between < 5% and > 60% within the area (D.J, Terlouw personal communication).

### Study design

Data were collected using a mixture of methods. An initial qualitative phase was conducted to explore community perceptions of anti-malarials and experiences of administering medications to children. This involved a series of focus group discussions (FGDs) and critical incidence interviews (CIIs). Results of the qualitative phase were then used to develop a structured questionnaire, which gathered data on experience of recent treatment and adherence to anti-malarials. This questionnaire was used during follow-up (FU) visits with caregivers whose child was participating in the parent trial, who had attended the study-site with malaria symptoms and was treated with either dispersible AL or DHA-PPQ.

### Qualitative data collection

FGDs and CIIs were conducted between September 2010 and February 2011. Qualitative data collection was conducted by four local fieldworkers who were trained in qualitative research methods, with a specific focus on conducting CIIs and FGDs and exploring issues around anti-malarial drug use. Two female fieldworkers (one facilitator and one note-taker) conducted FGDs with female participants and two male fieldworkers conducted FGDs with male participants. FGD participants were selected from among the community in which the parent trial was conducted. CII participants were identified by a continuous (‘rolling’) Malaria Indicator Survey (MIS) which involved visits to individuals’ homes and was conducted as part of the trial to monitor malaria prevalence in the area [29].

12 FGDs and 22 CIIs were conducted in the local language (Chichewa, although some Chisena was used in remote villages) (Table 1). Six initial FGDs were conducted followed by repeat FGDs with the same group to discuss the earlier findings and to clarify and probe issues further. FGDs gathered information about community knowledge and perceptions of medicines used to treat malaria and challenges associated with administering medications to children. Maximum variation purposive sampling was used to select FGD participants, ensuring that a range of perspectives were included [30,31]. FGD participants were selected by community advisory groups (CAGs) set up as part of the parent trial. Each participant had one or more children. This was not restricted by age of child, in order to capture the views of older members of the community, who may be important decision makers. FGDs were conducted in two village clusters, representing two diverse sub-sets of the population in the Chikhwawa district - those living around the commercial centre of the district, and those living in remote areas. Discussions held with CAGs during selection emphasized the importance of identifying ‘symbolically’ representative members of the community; criteria included selecting individuals with metal and straw roofs; those who lived nearer and further from the village centre; and those from different family groups. The team were aware of the likelihood that friends and relatives might be selected, however previously collected household wealth data suggested that within each sub-community, individuals were fairly homogenous in terms of possession ownership as a proxy for wealth [32]. Separate groups were conducted with younger women, older women and men of mixed ages.

**Table 1:** **Qualitative methods**

**Focus group discussions** ^**1**^	
Young women	**4**
Older women	**4**
Men	**4**
Total focus group discussions	12
**Critical incidence interviews**	
Women whose child experienced fever and attended a formal health facility	**12**
Women whose child experienced fever and did not attend a formal health facility	**10**
Total critical incidence interviews	22

^1^8 to 10 participants per group.

The 22 CIIs were conducted by one of the female fieldworkers. CIIs were used to explore participants’ experience of receiving and administering anti-malarials. The critical incident technique was first developed as a method of observing human behaviour in order to solve practical problems, where observed events were complete incidents with definite consequences [33]. This method enables the participant to develop a narrative that focuses around the particular influential event [34]. CII participants were caregivers of children under the age of five who were identified by the MIS as having had a febrile illness during the previous two weeks. The children had either received standard formulation AL at a health facility (with or without an RDT-based diagnosis) or had been treated by the survey team with dispersible AL following a positive rapid diagnostic test. CII participants were equally divided into those living less-than-15-minutes and greater-than-15-minutes walk from a health centre. Participants were asked about their experience of receiving and administering anti-malarials to their child during the recent febrile illness. Individuals midway through a treatment course were excluded.

Detailed topic guides were used to focus the CII and FGDs on the areas of interest and ensure all the relevant topics were covered [30]. However the fieldworkers were encouraged to use the topic guide flexibly, asking questions in any order and adding questions to gain more detail or explore topics of interest [35]. FGD Participants were asked to discuss: which household members are involved in administering anti-malarials to children; challenges experienced during administration; strategies for overcoming challenges; any known negative effects of various known anti-malarials; and how they identified and responded to different treatment outcomes. Draft topic guides were reviewed and refined with the fieldworkers before and after piloting. Two small pilot FGDs were held in villages close to the field site and pilot CIIs were held with three caregivers who had attended the facility for treatment. Voice recordings of pilot and all subsequent CIIs were reviewed on return from the field; translated transcripts of the pilot FGDs were discussed in detail, highlighting specific areas for encouragement and training. Transcripts of each subsequent FGD were reviewed in detail during preparation for repeat FGDs.

### Quantitative data collection

Results from analysis of the qualitative data were used to guide the development of a structured questionnaire to explore perceptions and utilization of anti-malarials in a larger group of participants. When participants in the parent trial attended the clinic and were treated with one of the two study drugs (AL or DHA-PPQ), they were invited to participate in a FU visit that would be conducted in their home. They were not made aware that this would involve a questionnaire survey, or that they would be questioned about their adherence. Individuals who had participated in a CII were excluded from the survey. FU visits were conducted by a fieldworker the day after treatment should have been completed, using a structured questionnaire delivered in the local language. The questionnaire consisted of four sections: basic demographics; medication and any instructions received at the clinic; the medication administration process; and drug package examination and adherence diary. Questions were designed to demonstrate an understanding of the types of issues experience when administering medication to young children, in order to gain confidence and improve accuracy of responses.

### Data analysis

Qualitative analysis examined in detail issues surrounding anti-malarial utilization. The team met after each qualitative data collection to review transcripts and discuss emerging themes. Qualitative data analysis was based on a framework approach [36]. The CIIs and FGDs were recorded, transcribed and translated into English, then imported into the qualitative analysis software QSR NVivo 9 for analysis. A broad index was developed based on themes emerging from the data and was refined throughout coding; the index was applied to code all data. Data were charted by summarizing and comparing data coded under different indexes and across different categories of participants, and developing explanations.

Quantitative data analysis was conducted to establish the proportion of children who were adherent to each ACT. Quantitative data was analysed using Stata version 10 (Stata Corp., College Station, TX). Individuals receiving AL were considered adherent if they reported administering tablets to their child twice daily for three days. Those receiving DHA-PPQ were considered adherent if they reported administering tablets once daily for three days. The proportion of individuals considered adherent in each group was calculated alongside odds ratios for the relationship between adherence and drug type. Child sex and age and maternal education were assessed for confounding and effect modification as they may influence adherence [18,20,23,37-40]. The proportion of caregivers who experienced challenges in administrating ACT to children and the proportion who received and understood instructions from the health facility were also explored,

### Ethics approval

Ethical approval was obtained from the College of Medicine Research and Ethics Committee, Malawi (P.04/09/783 and P.10/08/707) and the Liverpool School of Tropical Medicine Research Ethics Committee (09.07). Permission was also granted by village leaders. Sensitization campaigns were conducted as part of the parent trial to inform the communities about all aspects of the trial and affiliated studies. Fully informed written consent was obtained from all participants in the local language.

## Results

The results of the mixed-methods investigation is presented below. Initially quantitative data on overall adherence is presented. Qualitative findings then explore factors influencing ACT utilization, drawing on quantitative data where relevant.

### Overall adherence

218 individuals participated in the survey, no one declined (Table 2). Reported overall adherence was reasonably good: 88% (103/117) of those who received DHA-PPQ and 79% (80/101) of those who received AL adhered to the dosing schedule (Crude Odds Ratio: 1.93, 95% CI 0.92-4.06, P = 0.08). There was no difference in overall adherence according to child sex. There was a suggestion that, compared to children <24 months, older children were more adherent overall (24-35months: OR:1.40, 95% CI 0.63-3.09; 36-47months: 4.23, 95% CI 0.88-20.24, P = 0.07), but this did not differ between the two study drugs. Overall, level of maternal education did not affect adherence but there was a trend towards adherence being better with DHA-PPQ than with AL in the lower education groups (No Education: OR: 3.01, 95% CI 0.77-11.73, P = 0.10; Primary Education: OR 3.42, 95% CI 1.10-10.63, P = 0.03; Secondary Education: OR: 0.34, 95% CI 0.06-1.94, P = 0.21) (Table 3). Only one individual, who reported full adherence (n = 183), did not provide packaging. All other individuals reporting on adherence produced empty pill packaging. One individual who received DHA-PPQ (n = 117) and two who received AL (n = 101) reported not completing the treatment course but had no tablets remaining. All other participants who reported non-adherence had tablets remaining.

**Table 2:** **Survey participant characteristics according to whether they received Artemether-lumefantrine (AL) or Dihydroartemisinin-piperaquine (DHA-PPQ)**

	**Study arm**		
	**AL n(%)**	**DHA-PPQ n(%)**	**P** ^**a**^
**Child sex**			
Female	59 (58)	68 (58)	
Male	42 (42)	49 (42)	0.97
**Child age**			
<24 months	30 (30)	26 (22)	
24-35 months	53 (52)	76 (65)	
36-47 months	18 (18)	15 (13)	0.17
**Maternal education**			
None	25 (25)	35 (30)	
Primary	45 (45)	52 (44)	
Secondary +	31 (31)	30 (26)	0.60

^a^χ^2^/*χ* ^2^ test for trend.

**Table 3:** **Factors influencing adherence to the ACT Artemether-lumefantrine (LA) and Dihydroartemisinin-piperaquine (DHA-PPQ)**

	**Adherence to study drug**		
	**n (%)**	**Crude odds ratio (95% CI)**	**P** ^**a**^
**Anitmalarial**			
AL	80 (79)	1	
DHA-PPQ	103 (88)	1.93 (0.92-4.06)	0.08
**Child sex**			
Female	103 (81)	1	
Male	80 (88)	1.69 (0.78-3.68)	0.18
**Child age**			
<24 months	44 (79)	1	
24-35 months	108 (84)	1.40 (0.63-3.09)	
36-47 months	31 (94)	4.23 (0.88-20.24)	0.07
**Maternal education**			
None	49 (82)	1	
Primary	80 (83)	1.06 (0.46-2.44)	
Secondary +	54 (89)	1.73 (0.62-4.82)	0.30
**Adherence to AL vs DHA-PPQ Stratified by maternal education**			
No education	18 (72) vs 31 (89)	3.01 (0.77-11.73)	
Primary education	33 (73) vs 47 (90)	3.42 (1.10-10.63)	
Secondary education	29 (94) vs 25 (83)	0.35 (0.06-1.94)	
		Test of homogeneity:	P = 0.05

^a^χ^2^/*χ* ^2^ test for trend.

### Anti-malarial utilization

**Figure 1:**
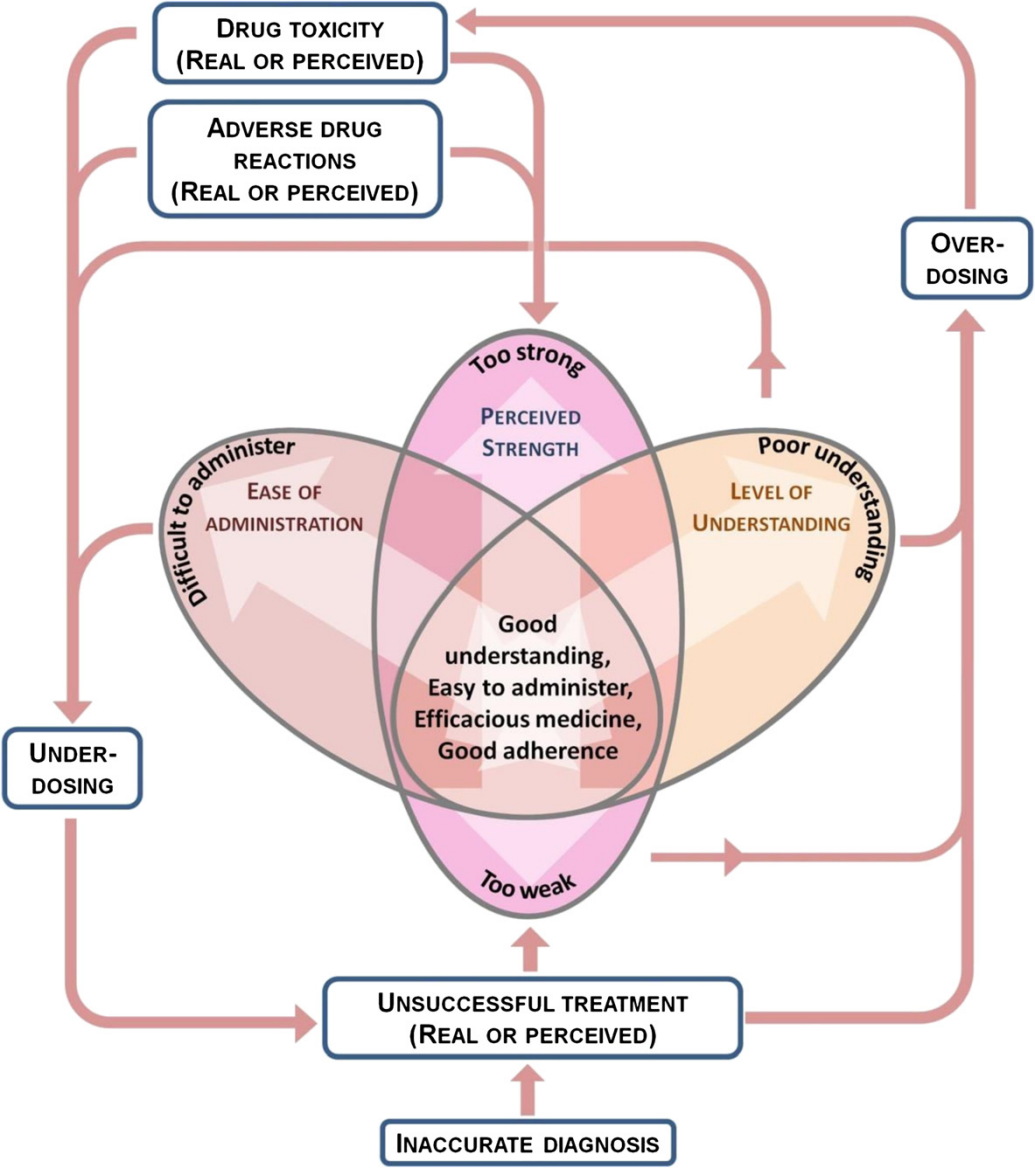
**Representation and anti-malarial utilization.** Perceptions and utilization of anti-malarials were found to be influenced by three main factors: perceived strength; ease of administration; and knowledge and understanding of appropriate dosing and causes of unsuccessful treatment. These factors were influenced by current or previous experience of treatment including treatment outcomes; and contributed to under-and overdosing. NB Drug toxicity may result from overdosing. Adverse events (AEs) may occur at recommended doses. AEs only influence individuals’ perceptions of strength and do not reflect the actual strength of the medication.

The main issues that emerged from the qualitative work were that perceptions and utilization of anti-malarials were determined by ease of administration; perceived strength; and understanding about the drug. Optimal adherence requires ACT that is easy to administer; perceived to be sufficiently, but not overly strong; is understood by caregivers both in terms of appropriate dosing and possible treatment effects and outcomes. The relationship between these factors and the influence of adverse drug reactions, drug toxicity and unsuccessful treatment is presented in Figure 1 and described below.

### Ease of administration

Adherence is only possible if caregivers are able to administer medication to the child. There was general agreement among FGD participants that the nature of the child determines the ease of administration:

*The difference comes in depending on the child’s mind, because there are some children who dislike drugs and there are some who easily receive drugs when they are sick.* (Younger women’s FGD, 08/10/10)

However, the bitter taste of medication in general was widely reported to be the cause of the problem in difficult children. Other reasons included a total loss of appetite, even for water, due to the illness and being tired of taking so many tablets.

Participants explained that methods used to manage difficult children varied according to their age. In general very young children were said to be unable to discern bad taste and were easier to administer medication to: a mother alone could hold an infant firmly between her legs, hold their noses or arms and force-feed the drug if necessary. She could also breastfeed immediately after to soothe them and prevent vomiting:

*The mother gives the drug and if she just stops there, the child will throw up, so what she does is to immediately offer the breast to the child.* (Men’s FGD, 07/10/10)

As children grew, it was reported that one or more other individuals may restrain them. It was frequently mentioned that older children might hold the drug in their mouth, later spitting it out. Participants explained that between the ages of five and 10 children could be bribed with small gifts to take tablets, but at this age they could also run away and hide. Older children were described as “too wise” and not easily bribed.

Male and older female FGD participants tended to describe physical methods, such as restraint, to ensure younger and older children take their medication. The younger women favoured threats for older children. Although other groups described threats, the women from HTR villages provided particularly dramatic descriptions, such as:

*“I will hit you, if you don’t take the medicine you’ll die”. So, the child is scared because she knows that if she doesn’t take the medicine, she’ll either die or be whipped.* (Young women’s FGD, 08/10/10)

It was apparent from the severity of the threats, and references to physical force, that the struggle between caregivers and children may contribute to long-term difficulty with adherence. Although infrequently recognized, a minority of participants highlighted that this conflict between caregivers and children leads to the child fearing hospital treatment. This relationship is further demonstrated by the following quote, where hospital treatment itself was the threat:

*Yes, “I will whip you, come on, swallow the drug or else I will tell the police to arrest you or the doctor will vaccinate you”.* (Young women’s FGD, 08/10/10)

A small number suggested talking nicely to children, reasoning with them, and using other positive methods such as mixing the medication with porridge or sugar to mask the taste.

A number of CII participants whose child received the standard formulation of AL reported that their child either struggled and refused to take the medication, spat it out, or cried as a result of taking it; by comparison no such comments were made by mothers of children who received dispersible AL. Use of physical force to ensure their child took the medication was described by half of the CII participants who had received standard AL, this was not reported by caregivers who received dispersible AL. One child who received dispersible AL spat out medication taken alongside AL, but did not spit out the AL. Two CII participants commented that their children did not complain about the taste of dispersible AL, whereas they usually complain that AL is bitter. One mother remarked that:

*This one smells so sweet that even a difficult child could cry for it.* (Young woman CII, 20/01/11)

However, dribbling, spilling and spitting remained a problem and were reported by 24% of FU participants in both study arms. The benefit of dissolving rather than crushing dispersible AL rarely featured in CIIs although once probed a couple of participants stated dispersible AL is easier to dissolve. 11% (11/100) of survey participants whose child received dispersible AL gave their children dispersible tablets to chew or swallow whole. One individual left the child to take medication themselves and did not observe treatment. In one FGD, the number of tablets was described as being a problem; participants stated the children get bored of taking so many tablets. Figure 1 shows how challenges in administration may lead to under-dosing, either through loss of medication (spillage etc.) or reduced adherence.

### Perceived strength

The perceived strength of anti-malarials emerged as a major theme. Figure 1 shows how the spectrum of views ranged from ‘too weak’ to ‘too strong’ with a small number of individuals at both ends, but most described both anti-malarials as good. In general, male participants voiced more negative views:

*Let’s be frank,****AL****is not a strong drug.* (Men’s FGD, 06/10/10)

Women, especially in HTR villages, were more positive.

Perceptions of medication strength had direct implications on adherence; individuals reported deliberately altering the dosing schedule in accordance with the perceived strength. In one FGD, participants explained that lack of trust in the strength of anti-malarials with consequent illness progression caused some individuals to give an over-dose in order to speed up the process:

Respondent 1: *You may be told to give one tablet of AL and half a tablet of paracetamol in the morning and in the evening, but only paracetamol at noon. We are clearly told how to do it and yet when we come back home, we think it is a joke and we just administer AL in the morning, at noon and in the evening.*

Respondent 2: *To do it fast…*

Interviewer: …*What do you mean by doing it fast?*

Respondent 1: *To heal fast.* (Older women’s FGD, 08/10/10)

At the other end of the spectrum, one survey participant who received AL stated she deliberately withheld tablets because the full dose would be too strong for the child.

### Understanding, knowledge and clarity of instructions

Almost all survey participants reported having received instructions on dosing at the health facility (99%, 215/218), and most reported having understood these instructions (98%, 213/215). There was general understanding of the importance of following the correct dose schedule and this positively influenced adherence:

*…we don’t stop it when we see that the child is rejoicing but we administer the entire dosage as instructed by the doctor. It isn’t proper to give the drug to another child. He has to complete the dosage prescribed to him. Each patient must have his own dosage.* (Older women’s FGD, 08/10/10)

Some individuals understood the potential impact of poor adherence on perceptions of anti-malarials:

*When you don’t follow the instructions, then you tend to think that the drug isn’t powerful enough, but when you are told to take the drug in the morning, at noon and in the evening, you must do so, if not, you won’t be cured.* (Men’s FGD, 06/10/10)

A number of individuals understood that unsuccessful treatment as a result of skipping doses, taking tablets at the incorrect time or failing to take AL with food may lead to negative perceptions of otherwise good medications.

Figure 1 shows how poor understanding of the dosing schedule and possible treatment outcomes was linked to both over- and under-dosing. Despite the importance placed on followed the correct dosing schedule, there were knowledge gaps regarding the schedule itself. Taking the tablets over a shorter period than recommended with consequent over-dosing, specifically taking AL morning, noon and evening, was frequently mentioned throughout qualitative data collection. This was supported by the quantitative data: 7% (7/101) of FU participants whose child received AL reported giving tablets three times daily rather than twice, and 3% (4/117) who received DHA-PPQ gave tablets twice daily rather than once. Messages, or interpretation of messages, received at the health facility contributed to inappropriate timing of doses. Participants explained that the first tablet is taken in the clinic, and the second as soon as they get home:

*We are advised to administer the drug as soon as we return home, so when we reach home, we take the drug and administer it to the child* (Older women’s FGD, 08/10/10)

For those living nearer to the clinic, this may be a short time interval, and some described taking the tablets within a couple of hours of each other, resulting in overdosing:

*He takes the drug at 10 in the morning and again at 12 O’clock and finally in the evening(Men’s FGD, 06/10/10)*

Some instructions were well understood: most individuals agreed that if the child vomited or spat out the medication, then another dose should be given, as instructed by the clinic. However there was a general misunderstanding that there was no need to go back to clinic to replace this dose, and this may lead to under-dosing. Vomiting was infrequently reported by household survey participants; 5.5% (12/218) of children had vomited following recent treatment; ten caregivers did nothing, one returned to the hospital, and one gave another tablet without replacing the dose. This contrast between stated understanding and actual behaviour suggests that other factors determine responses to vomiting.

Participants were found to fill in gaps in their understanding by developing theories to explain unsuccessful treatment and adverse events. Lack of resolution of symptoms following treatment and re-occurring illness were frequent complaints. These, along with the less frequently reported increases in symptoms or additional symptoms following the start of anti-malarial treatment, were widely interpreted as a lack of compatibility between the drug and the child’s body or “blood”:

*For some, it is not a suitable drug because when you administer AL to them, the fever gets worse, for others it is a suitable drug.* (Young women’s FGD, 11/10/10)

Both FGD and CII participants described how particular medication works or does not work well consistently in their child; the focus of these comments was the match between the child and the drug, rather than between the drug and the cause of illness.

Such experiences caused some participants to avoid returning to the health facility, for fear of receiving the same drug, and led others to request clinicians prescribe alternative medication. Participants also understood that unsuccessful treatment could be a sign that the child’s fever was not caused by malaria.

## Discussion

Measurement of adherence is extremely challenging due to the lack of an accurate biomarker and the influence of study design on participants’ behaviour. This study draws on the strengths of multiple methodologies to explore experiences of using ACT. Qualitative methods, which allow for the development of rapport with participants, were used to explore experiences of anti-malarial use in detail, both generally in FGDs, and during a recent course of treatment in CII. Results were used to develop a context specific questionnaire, which explored a wide range of aspects of medication use across a larger population.

Participants had generally positive perceptions of both study drugs, in line with the observed high level of adherence. Participants demonstrated a good understanding of the importance of correct dosing; however there was some confusion over specific details of the dosing schedule. Non-adherence included over-dosing through taking all doses over a shorter period than intended, taking a reduced dose over a longer period of time and failing to complete the entire treatment course, all of which have been previously reported [18,41,42]. Under-dosing potentially leads to treatment failure and may contribute to the spread of anti-malarial drug resistance. While adverse events can occur at the recommended dosage, overdosing may lead to drug toxicity and contribute to poor perceptions of available anti-malarials and discourage appropriate treatment seeking. Health workers involved in dispensing anti-malarials should be made aware of these issues and encouraged to target these knowledge gaps when providing instructions at the health facility.

Most FU participants reported having understood instructions received at the health facility, in this case provided within a study setting. Quality of instructions has been found to significantly influence adherence [10,20,43]. A Nigerian study showed similar findings of participants taking medication three times a day for two days or once daily for six days, rather than twice daily for three days and attributed this to misunderstanding of the drawings that were used as instructions [15]. In the current study, participants placed importance on following instructions given at the health facility, indicating the considerable potential impact of clarifying and reinforcing such messages. Combining verbal instructions with the use of pictorial instructions has been linked to improved adherence in Malawi [20]: health workers should be encouraged to make use of the pictorial instructions present on the drug packaging. In addition, further optimization of the packaging design should also be considered. Whilst qualitative data collection suggested health education messages regarding the importance of giving another dose following vomiting were getting through, the quantitative data indicated this was not being put into practice.

Individuals frequently interpreted poor treatment outcomes and adverse events as lack of compatibility between the child and the medication. This supports findings that individuals make decisions about the choice of treatments based on the perceived effectiveness of the medication for a particular set of symptoms in a specific illness episode, rather than taking a ‘one-size-fits-all’ perspective [25]. These results emphasize the importance of ensuring both caregivers and health facility staff have a good understanding of reasons for poor treatment outcomes, possible adverse events, and appropriate responses to severe adverse events, in order to prevent community members developing theories to explain such events.

The bitter taste of medications was said to be a general problem in administering medication to children. Challenges in administration contributed to caregivers using threats and physical restraint during delivery of anti-malarials. This study suggests there were some benefits of the improved dispersible flavoured formulation in decreasing the conflict associated with administering medications, but dribbling, spilling and spitting remained a problem in both study arms. Alternative formulations and delivery systems need to be considered in order to improve the administration of anti-malarials for all involved, to reduce medication loss, and improve adherence. Furthermore, the dispersible formulation is only currently available in packages suitable for the youngest age groups, whereas participants reported most difficulty in administering medication to older children; highlighting the importance of considering the role of alternative formulations for older children.

Despite the improved taste and solubility of dispersible AL, our findings suggest that adherence was greater to DHA-PPQ. This seemed largely influenced by the easier schedule of DHA-PPQ. Both drugs were assessed within the context of a safety and effectiveness trial, with treatment provided by trained study clinicians, although previous experience with AL may have influenced adherence. The DHA-PPQ had trial packaging with no pictorial instructions, so correct understanding depended on instructions received during dispensing and not on the individuals’ interpretation of pictures.

### Methodological issues

The level of reported adherence in this study may be higher than that of the general population due to participation in the broader clinical effectiveness trial and associated greater community knowledge and the dispensing of medication by trained study clinicians, who may have placed greater importance on adhering to instructions; and participants’ awareness of possible home visits. It is possible that participants exaggerated the extent to which they were adherent due to knowledge of the desired behaviour. Efforts were made to avoid this: questionnaire development drew on information gained during the qualitative phase of research, to frame questions that explored in detail the experiences of administering medication to the child. This included probing into any challenges experienced, in order to develop rapport and provide a context for adherence, before completing the adherence diary. In addition, drug packaging was checked prior to questioning on adherence and the high correlation between reported adherence and package checks suggests the accuracy of reports.

## Conclusion

Despite high overall reported adherence, caregivers experience great difficulty in administering medication to children, the consequences of which include conflict between the child and caregiver; loss of medication; and reduced adherence. While the sweet taste of dispersible AL may have reduced difficulties, sub-optimal dosing due to medication loss remained a problem and our findings suggest that overall adherence was greater among those receiving DHA-PPQ, which had a once-daily regimen and so required fewer doses. A lack of understanding of the correct dosing also contributed to under- and over-dosing. Key areas for improvement include: strengthening health education messages around the importance of correct dosing to parents; providing a combination of clear pictorial and verbal instructions during dispensing; and explaining the consequences of under and over-dosing, alongside appropriate responses to and reporting of possible adverse events. Further optimization of anti-malarial adherence in children requires the development of anti-malarials with pharmacological properties that allow user-friendly administration and simplified dosing schedules.

## Abbreviations

AL: Artemether-lumefantrine
DHA-PPQ: Dihydroartemisinin-piperaquine
ACT: Artemisinin-based combination therapy
SP: Sulphadoxine-pyrimethamine
FGDs: Focus group discussions
CIIs: Critical incident interviews
CAGs: Community advisory groups
OR: Odds ratio
